# Open chromatin dynamics in prosensory cells of the embryonic mouse cochlea

**DOI:** 10.1038/s41598-019-45515-2

**Published:** 2019-06-21

**Authors:** Brent A. Wilkerson, Alex D. Chitsazan, Leah S. VandenBosch, Matthew S. Wilken, Thomas A. Reh, Olivia Bermingham-McDonogh

**Affiliations:** 1 Department of Biological Structure, University of Washington, Box 357420, Seattle, WA 98195 USA; 2 Institute for Stem Cells and Regenerative Medicine, University of Washington, Box 358056, Seattle, WA 98109 USA; 3 Department of Biochemistry, University of Washington, Seattle, WA 98195 USA; 4 Molecular and Cellular Biology Program, University of Washington, Seattle, WA USA; 5 Present Address: Altius Institute for Biomedical Sciences, Seattle, WA 98121 USA

**Keywords:** Developmental neurogenesis, Cochlea

## Abstract

Hearing loss is often due to the absence or the degeneration of hair cells in the cochlea. Understanding the mechanisms regulating the generation of hair cells may therefore lead to better treatments for hearing disorders. To elucidate the transcriptional control mechanisms specifying the progenitor cells (i.e. prosensory cells) that generate the hair cells and support cells critical for hearing function, we compared chromatin accessibility using ATAC-seq in sorted prosensory cells (Sox2-EGFP^+^) and surrounding cells (Sox2-EGFP^−^) from E12, E14.5 and E16 cochlear ducts. In Sox2-EGFP^+^, we find greater accessibility in and near genes restricted in expression to the prosensory region of the cochlear duct including *Sox2*, *Isl1*, *Eya1* and *Pou4f3*. Furthermore, we find significant enrichment for the consensus binding sites of Sox2, Six1 and Gata3—transcription factors required for prosensory development—in the open chromatin regions. Over 2,200 regions displayed differential accessibility with developmental time in Sox2-EGFP^+^ cells, with most changes in the E12-14.5 window. Open chromatin regions detected in Sox2-EGFP^+^ cells map to over 48,000 orthologous regions in the human genome that include regions in genes linked to deafness. Our results reveal a dynamic landscape of open chromatin in prosensory cells with potential implications for cochlear development and disease.

## Introduction

Hearing is mediated by a specialized sensory epithelium, the organ of Corti, within the cochlea of the inner ear. In the organ of Corti, rows of hair cells interdigitated with support cells sense sound^1^. Hair cell loss or dysfunction underlies most sensorineural hearing loss. Hearing loss is broadly affected by genetics and perhaps also by epigenetics. Genetic factors contribute to 68% of hearing loss at birth—affecting 1–2 newborns per 1000—as well as to progressive hearing loss in 2.7 children per 1000 by adolescence^2^. In addition to congenital hearing loss, significant genetic contributors have been identified for all major acquired forms of hearing loss including presbycusis^3–8^, noise-induced hearing loss^9–18^ and ototoxic medication-induced hearing loss^19–21^. Hearing loss-causing variants in 153 genes have been reported including variants that map to introns and other noncoding regions of the genome^22^. Detailed characterization of the regulatory genome in the inner ear will likely lead to a better understanding of how genetic differences influence the etiology of congenital and acquired hearing loss.

In other tissues and cell types, concerted efforts have characterized a wide range of gene regulatory elements by eQTL analysis^23^ and by genome-wide sequencing of open chromatin, histone-mark enrichment, transcription factor-binding and other epigenomic features^24^. Tissue- and cell-specific ‘maps’ of regulatory elements are now crucial resources for systems studies of gene regulation and gene networks. Such studies promise greater understanding of the function of genetic variants in noncoding regions and the identification of enhancers suitable for the experimental and therapeutic goals of targeted transgenesis.

Despite the vast potential utility of mapping the regulatory genome of inner ear cell types, little progress has been made in this regard. Elements having enhancer activity in otic tissues have been identified based on evolutionary conservation^25^ and on histone marks associated with gene-activation in other tissues^26^. Recent studies detected enhancers using ATAC-seq and MethylC-seq in whole mouse cochlea^27,28^. Mapping of open chromatin in specific cell types of the cochlea using ATAC-seq can further elucidate the regulatory genome and related gene networks of cochlear development.

Hair and support cells differentiate from a region of the floor of the cochlear duct called the prosensory domain. The cells of the prosensory domain exit the mitotic cycle between E12 and E16 in an apical to base sweep^29,30^. They begin their differentiation as hair cells or support cells within a few days first at the base and lastly at the apex^29–33^.

The regulation of specification of prosensory cells involves Notch-, Bmp- and Fgf-signaling^34–45^ as well as the transcription factors Gata3, Sox2 and Six1^46–53^. Differentiation of support cells requires Fgf-signaling and Notch-signaling^54,55^; differentiation of hair cells requires Wnt-signaling, Atoh1 and Pou4f3^56–58^. To better understand the interactions among these signaling molecules and transcriptional regulation of cochlear development, we used ATAC-seq to map the open chromatin regions in cells of the prosensory domain (Sox2-EGFP^+^) as well as the surrounding nonsensory cells (Sox2-EGFP^−^) isolated from the embryonic mouse cochlear duct.

This approach detected over 65,000 open chromatin regions in Sox2-EGFP^+^ cells from embryonic cochlear duct, including many regions mapping to known and putative gene regulatory regions. Motif enrichment analysis of these open chromatin regions identifies classes of transcription factors not previously implicated in inner ear development and provides strong evidence for a prosensory-specific landscape of open chromatin. Differential accessibility analysis across three developmental stages elucidated epigenetic dynamics in prosensory cell development. These datasets not only provide insights into mechanisms of prosensory development in the cochlea, but also represent a significant resource for the field of epigenomic research in the inner ear and future mechanistic studies of *cis*-regulation of deafness genes.

## Results

### Sox2-EGFP expression in prosensory cells of the embryonic cochlea

**Figure 1:**
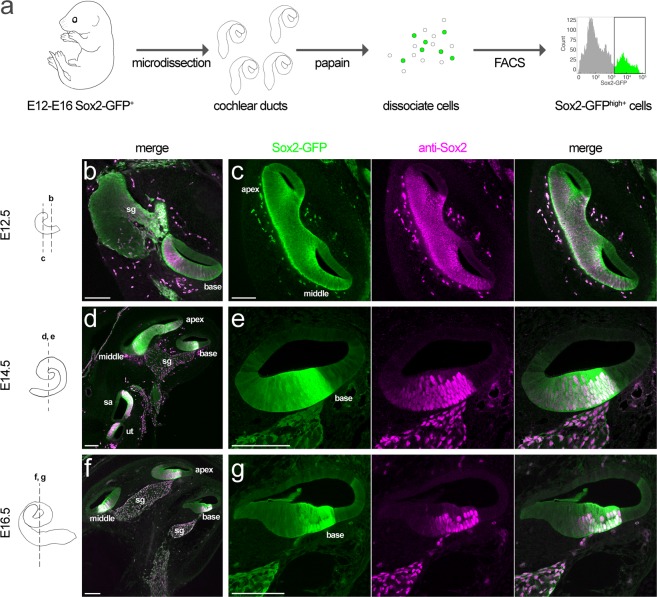
Sox2-EGFP expression in prosensory cells of the embryonic cochlea. (**a**) Shows the tissue isolation and FACS pipeline used to generate the cells. (**b**–**f**) Show Sox2-EGFP expression (*green*) in vibratome sections of cochlea at the indicated stages of embryonic development. Sox2 immunofluorescence (*magenta*) demonstrates both the prosensory cells in the cochlear duct and the glia of the spiral ganglion. Note that Sox2-EGFP expression corresponds to Sox2 immunofluorescence and that Sox2-EGFP developmental dynamics mirror those of endogenous Sox2 expression. For example, the Sox2^+^/Sox2-EGFP^+^ field of cells in the floor of the cochlear duct narrows between E12.5-16.5—first at the base of the duct, then apically (compare **b**,**d** and **f**) and first at the lateral side of the duct, then medially (compare **c**, **e** and **g**). The localization of Sox2-EGFP expression shown here is representative of that in at least three temporal bones. Scale bars = 100 μm. *sg*, spiral ganglion; *sa*, saccule; *ut*, utricle.

To selectively characterize the accessible chromatin in prosensory cells in the developing cochlea, we carried out ATAC-seq on FACS isolated EGFP^+^ cells from Sox2-EGFP mice, a strain having EGFP knocked-into the *Sox2* coding region^59^. To determine whether EGFP specifically marks cochlear prosensory cells in heterozygous Sox2-EGFP mice, Sox2-EGFP expression in prosensory cells was compared to Sox2 immunofluorescence at several developmental stages along the cochlea spiral in vibratome sections of E12-16 temporal bones. Similar to endogenous Sox2 expression, the highest level of Sox2-EGFP immunofluorescence is evident in the prosensory cells of the cochlear duct as well as the glia of the spiral ganglion in the E12, E14 and E16 cochleae (Fig. 1). In the following study, cochlear ducts were carefully dissected to remove developing spiral ganglion neurons and associated glia.

Developmental dynamics in Sox2-EGFP expression mirror those of endogenous Sox2 expression; the cells expressing the highest level of EGFP (Sox2-EGFP^high+^) correspond to the Sox2^+^ immunolabeled cells. For example, the anti-Sox2^+^/Sox2-EGFP^high+^ domain initially occupies the entire floor of the cochlear duct (Fig. 1b,c) and becomes progressively restricted between E12.5-16.5, first at the base of the duct, then apically (compare Fig. 1b,d,f). Furthermore, the anti-Sox2^+^/Sox2-EGFP^high+^ field of cells in the floor of the cochlear duct narrows first at the lateral side of the duct, then medially (compare Fig. 1c,e,g). Laterally, p75/Ngfr immunofluorescence in Claudius cells flanks anti-Sox2^+^/Sox2-EGFP^high+^ expression at E16 (Supplementary Fig. 1). Similar to the embryonic cochlea, Sox2-EGFP^high+^ corresponds with endogenous Sox2 in E12-16 utricle, saccule and cristae (Supplementary Figs 2 and 3).

Although low and intermediate levels of Sox2-EGFP were observable in some nonsensory cells of the cochlear duct, these findings support a strategy to isolate Sox2-EGFP^high+^ prosensory cells from dissociated cochlear duct cells by gating for cells having the highest Sox2-EGFP signal in FACS (Fig. 1a). FACS demonstrated that Sox2-EGFP^high+^ cells represent ~70% of the E12 cochlear duct, ~50% of the E14.5 cochlear duct and ~25% of the E16 cochlear duct (Supplementary Fig. 4), which reflects the size of the Sox2-EGFP^high+^ regions observed in midmodiolar sections (Fig. 1).

### Open chromatin regions in Sox2-EGFP^high+^ cells of the embryonic cochlear duct map to genes and gene-regulatory regions including known otic enhancers

**Figure 2:**
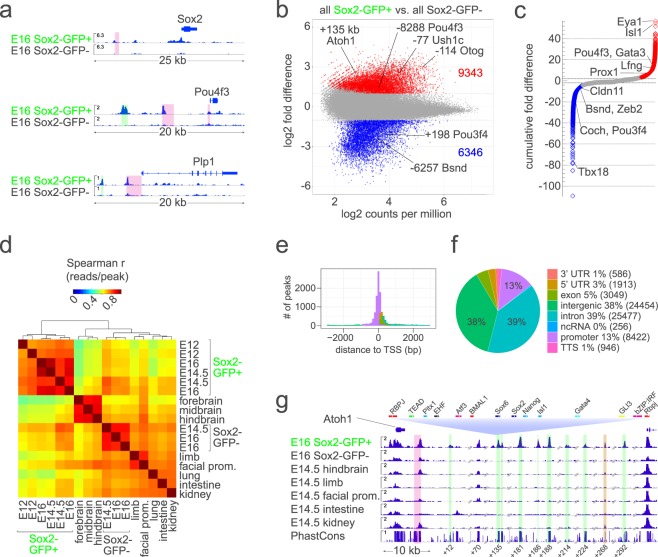
ATAC-seq detects gene regulatory features that include known otic enhancers in Sox2-EGFP^*high*+^ cells of the embryonic cochlear duct. (**a**) Shows ATAC-seq signal in E16 Sox2-EGFP^*high*+^ cochlear cells relative to that in E16 Sox2-EGFP^−^ cochlear cells collected in parallel at the *Sox2*, *Pou4f3*, and *Plp1* loci. Highlighted in *pink* are known enhancer regions. Highlighted in *green* are novel open chromatin regions detected only in Sox2-EGFP^*high*+^ cells of the embryonic cochlear duct. (**b**) Shows fold difference relative to the normalized read counts for ATAC-seq peaks determined to be significantly increased (*red*), significantly decreased (*blue*) or unchanged (*grey*) in Sox2-EGFP^*high*+^ cells from E12-16 cochlear duct versus Sox2-EGFP^−^ cells from E14.5-16 cochlear duct. (**c**) Shows the cumulative fold differences (i.e. the sum of the fold differences of all peaks nearest to each gene) in accessibility of each gene in Sox2-EGFP^*high*+^ cells from E12-16 cochlear duct versus Sox2-EGFP^−^ cells from E14.5-16 cochlear duct. In the highlighted genes, accessibility corresponds to known differential patterns of gene expression. (**d**) shows a clustered Spearman correlation matrix of the reads in peaks from ATAC-seq of FACS-sorted cochlear cells and of various E14.5 tissues. Note that reads in cochlear ATAC-seq samples are highly correlated. (**e**) Shows the frequency of ATAC-seq peaks relative to the distance to transcription start sites. (**f**) Shows the percentages of all replicated peaks detected in Sox2-EGFP^*high*+^ cells of the E12-16 cochlear duct mapping to the indicated genomic features. (**g**) Shows ATAC-seq signal at the *Atoh1* locus in E16 Sox2-EGFP^*high*+^ cochlear cells aligned to that in E16 Sox2-EGFP^−^ cochlear cells collected in parallel; several other E14.5 tissues, and PhastCons 30-way vertebrate conservation. As an example of transcription factor binding motifs in peaks, we show those predicted in the +135 kb peak. Highlighted in *pink* is the known 3′ enhancer. Highlighted in *green* are 7 open chromatin regions specific to Sox2-EGFP^*high*+^ cells of the embryonic cochlear duct downstream of *Atoh1*. Highlighted in *gold* is a region that increased in accessibility in E14.5 vs. E12 Sox2-EGFP^*high*+^ cells of the embryonic cochlear duct. Transcription factor families having similar bindings motifs are color-coded. Highest scoring members of each family are indicated.

We carried out ATAC-seq on FACS purified Sox2-EGFP^high+^ and Sox2-EGFP^−^ cells, we sequenced and mapped the reads, and then identified peaks with MACS2^60^. We found >65,000 replicated peaks in the E12-16 Sox2-EGFP^*high*+^ cochlear duct cells (e.g. Fig. 2a, Supplementary Data 1). To determine whether ATAC-seq detects known otic enhancers, we curated a comprehensive list of otic enhancers and promoters (i.e. driving gene expression *in vivo*) from published literature on all species and the VISTA Enhancer database^61^, then compared the enhancers and promoters to ATAC-seq peaks detected in Sox2-EGFP^*high*+^ and Sox2-EGFP^−^ cells (Supplementary Data 2). ATAC-seq peaks mapped to 41 of the 56 enhancers and promoters that were previously reported to have otic activity, including those of *Sox2*, *Pou4f3, Plp1* and *Atoh1* (Fig. 2a,g and Supplementary Data 2).

To better resolve quantitative differences in accessibility at the peaks found in Sox2-EGFP^high+^ and Sox2-EGFP^−^ cells, we carried out a differential accessibility analysis using edgeR^62^ that calculates significance and fold-differences based on normalized reads per peak in replicate samples. We detected 9,343 peaks that show significantly greater accessibility in Sox2-EGFP^high+^ cells than in surrounding Sox2-EGFP^−^ cells and 6346 peaks showing significantly less accessibility in Sox2-EGFP^high+^ cells than in Sox2-EGFP^−^ cells (Fig. 2b, Supplementary Data 3). Several genes known to be differentially expressed in prosensory and nonsensory cells such as *Isl1*^63^ and *Bsnd*^64^, respectively, showed corresponding differences in accessibility (Fig. 2c). This can be seen in Fig. 2c as the cumulative fold difference in accessibility of all peaks nearest to each gene. We further confirmed the efficacy and specificity of ATAC-seq by comparing the Sox2-EGFP^high+^ peaks with previously reported unmethylated regions and low methylated regions of the E16.5 cochlear sensory epithelium^65^ (Supplementary Fig. 5). We find that most ATAC-Seq peaks in cochear duct cells correspond to unmethylated and low methylated regions.

To further test for the specificity of the peaks in the Sox2-EGFP^high+^ cells, we carried out a correlation analysis of reads present per ATAC peak in E12-16 ATAC-seq samples of Sox2-EGFP^*high*+^ cochlear duct cells to reads present per ATAC peak in ATAC-seq samples from other tissues (Fig. 2d). We combined peaks from several studies called by MACS2 (>90,000) and examined the correlation of reads in peaks in every sample. We found high correlation among cochlear Sox2-EGFP^high+^ samples, highlighting the unique epigenomic landscape of prosensory cells. Similar to accessible chromatin in other tissues^66,67^, the Sox2-EGFP^high+^ ATAC-seq peaks increased in frequency near transcription start sites (Fig. 2e) and primarily mapped to introns and potential cis-regulatory intragenic regions (Fig. 2f and Supplementary Fig. 6). Furthermore, replicated peaks detected in E12-16 Sox2-EGFP^*high*+^ cochlear duct cells showed over 30-fold enrichment for CpG islands, 5′ untranslated regions and promoters (13,225, 13,069 and 14,026 peaks, respectively; Supplementary Fig. 6). Of the >65,000 peaks called in the Sox2-EGFP^high+^ cells, over 29,000 peaks were detected only in Sox2-EGFP^*high*+^ cells of the embryonic cochlear duct. These peaks were not present in E14.5-16 Sox2-EGFP^−^ cochlear duct cells, or in the other embryonic tissues we analyzed, and include regions near known regulators of inner ear development. As an example of this Fig. 2g depicts the Atoh1 locus and these open chromatin regions are highlighted in green (coordinates in Supplementary Data 1). We did not successfully prepare ATAC-seq libraries from E12 Sox2-GFP^−^ cells, which are a small percentage of the cochlear duct (Supplementary Fig. 4), so we did not include this stage of Sox2-EGFP^−^ cells in our analysis.

### Differential enrichment of transcription factor-binding motifs in Sox2-EGFP^high+^ cells of the embryonic cochlear duct

**Figure 3:**
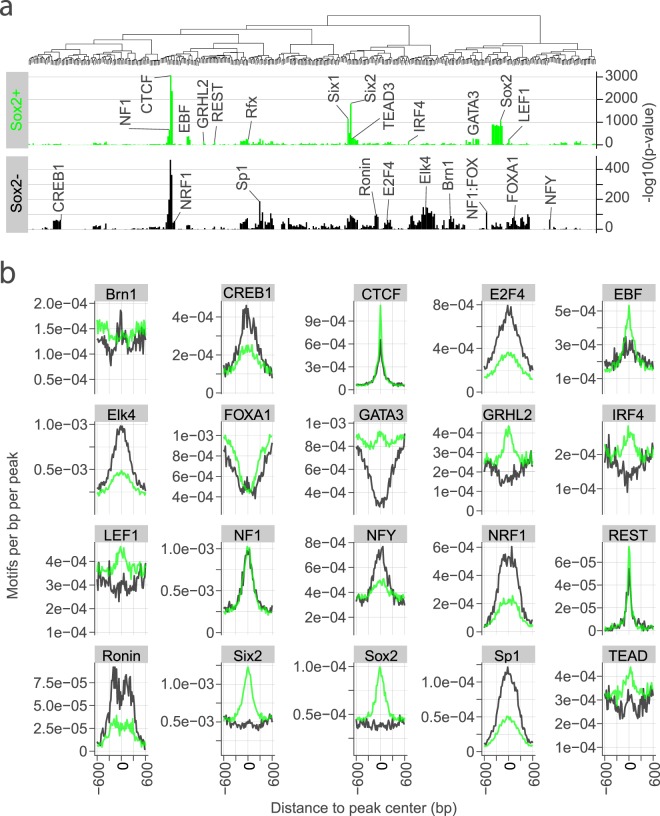
Motif enrichment in open chromatin regions in Sox2-EGFP^*high*+^ cells of the embryonic cochlear duct. (**a**) Plots qualitative differences in motif enrichment in Sox2-EGFP^*high*+^ cells from E12-16 cochlear duct and Sox2-EGFP^−^ cells from E14.5-16 cochlear duct as −log10 of the p-value in replicated peaks. Motifs are clustered based on similarity. The most highly enriched members of transcription factor families/clusters are selectively labeled based on differential enrichment in the samples. Differences in enrichment might be indicative of context-specific transcription factor activity. Scales in (**a**) vary due to differences in peak number and coverage. (**b**) Plots quantitative differences in enrichment of representative motifs as the frequency of motifs relative to peak center. Note that high enrichment (**a**) often corresponds to greater central localization in peaks (**b**).

To better understand the regulatory circuits controlling cochlear development in the prosensory region, we carried out an analysis of transcription factor binding motifs in the Sox2-EGFP^high+^ peaks. Motif enrichment in replicated ATAC-seq peaks detected in Sox2-EGFP^high+^ cells of the E12-16 cochlea duct and in Sox2-EGFP^−^ cells of the E14.5-16 cochlea duct were compared using the p-value of enrichment for all motifs in the HOMER library (Fig. 3a) and the frequency of motifs (Fig. 3b). To group similar motifs in Fig. 3a, motifs were clustered based on the correlation of motif matrices (Supplementary Fig. 7). Scales vary in Fig. 3b with differences in motif abundance.

**Figure 4:**
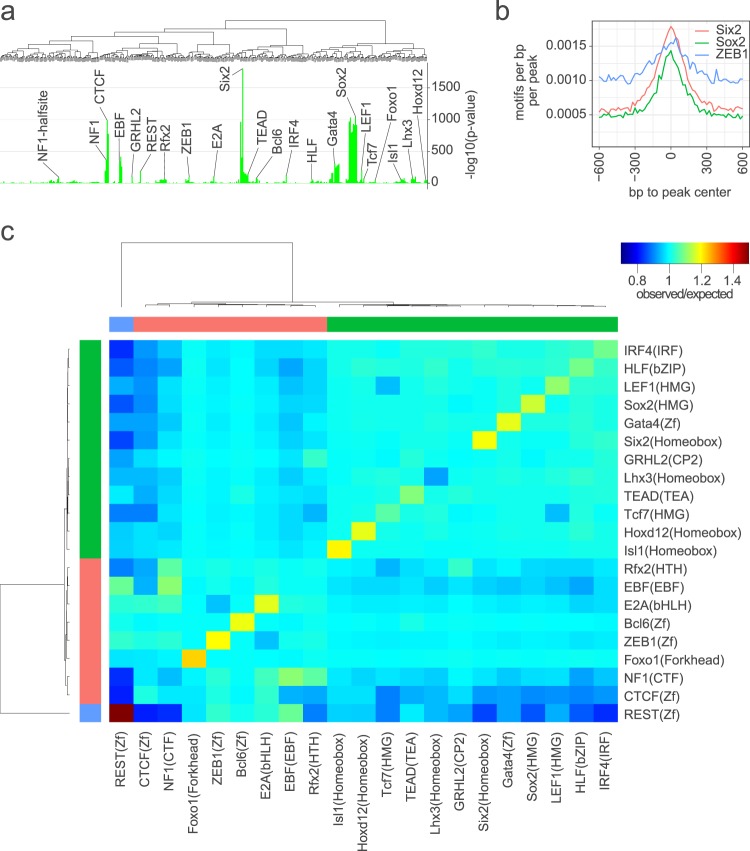
Motif enrichment and co-occurrence in prosensory-specific open chromatin regions of the embryonic cochlear duct. (**a**) Shows the motif enrichment as -log10 of the p-value in the subset of replicated peaks detected only in Sox2-EGFP^*high*+^ cells from E12-16 cochlear duct (i.e., not detected in Sox2-EGFP- cells or other ENCODE datasets examined). (**b**) Plots quantitative differences in enrichment of representative motifs as the frequency of motifs relative to peak center. (**c**) Plots clustered matrix of the ratio of the observed frequency of co-occurrence of each combination of enriched motifs in the prosensory-specific peaks relative to the expected frequency of co-occurrence.

Open chromatin regions in Sox2-EGFP^*high*+^ cochlear duct cells showed greater enrichment for motifs corresponding to factors in the Six, Sox, Gata, Ebf and Tead families as well as motifs for Grhl2, Lef1, Irf4 and Rest (Fig. 3, Supplementary Data 4). Motif enrichment analysis in the ‘prosensory-specific’ subset (i.e., the 29,000 peaks detected only in Sox2-EGFP^*high*+^ cells of the embryonic cochlear duct) was similar to that in peaks of Sox2-EGFP^*high*+^ cells (Fig. 4a,b) with some differences. For example, ‘prosensory-specific’ ATAC regions showed 2–3-fold enrichment for Six2 and Sox2 motifs and enrichment also for the Zeb1 motif (Fig. 4b). Relative to that in Sox2-EGFP^*high*+^ cells, open chromatin regions in Sox2-EGFP^−^ cochlear duct cells showed greater enrichment for motifs corresponding to the factors in the Forkhead, E2f, Ets, Sp/Klf, bZip and Pou families, as well as Nfy, Nrf1 and Ronin. Ctcf, Rfx and Nf1 were similarly enriched for in Sox2-EGFP^*high*+^ and Sox2-EGFP^−^ cells.

To look for evidence of combinatorial transcription factor activity in prosensory open chromatin, we analyzed motif co-occurrence in the ‘prosensory-specific’ subset (i.e., the 29,000 peaks detected only in Sox2-EGFP^*high*+^ cells of the embryonic cochlear duct. Examining the ratio of observed co-occurrence in the prosensory-specific peaks, relative to baseline co-occurrence in the genome, highlights that some combinations of motifs occur in prosensory open chromatin regions at frequencies greater than random chance (Fig. 4c and Supplementary Data 7). Motif co-occurrence analysis showed that many motifs occur multiple times in the same open chromatin regions (see diagonal in Fig. 4c).

### Chromatin dynamics in Sox2-EGFP^high+^ cells of the embryonic cochlear duct

**Figure 5:**
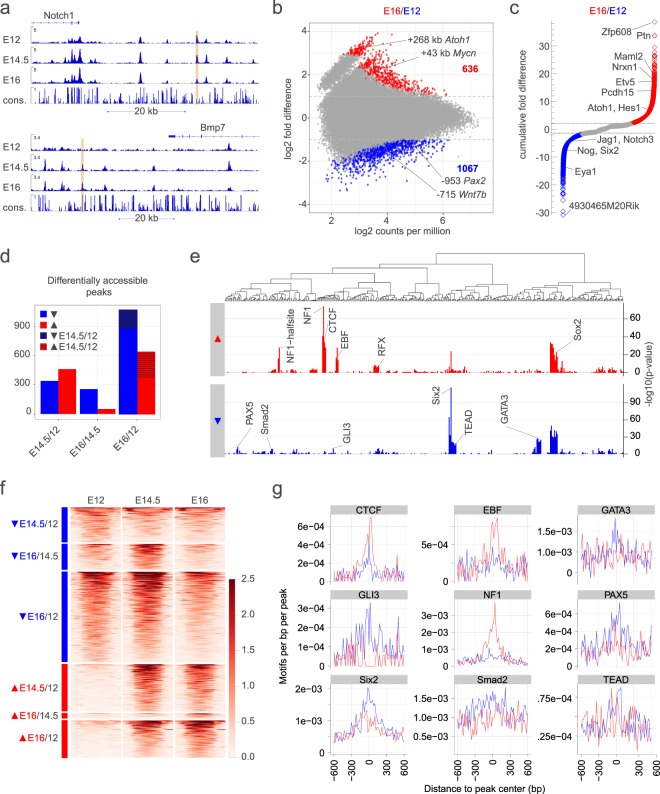
Developmentally-dynamic open chromatin in Sox2-EGFP^*high*+^ cells of the embryonic cochlear duct. (**a**) Shows examples of developmentally dynamic ATAC-seq peaks (*gold*) in Sox2-EGFP^*high*+^ cells of the E12-16 cochlear duct at the indicated loci. (**b**) Shows the fold difference relative to the normalized read counts for ATAC-seq peaks determined to be significantly increased (*red*), significantly decreased (*blue*) or unchanged (*grey*) in Sox2-EGFP^*high*+^ cells from E16 cochlear duct versus those from E12 cochlear duct. (**c**) Shows the cumulative fold differences (i.e. the sum of the fold differences of all peaks nearest to each gene) in accessibility of each gene in Sox2-EGFP^*high*+^ cells from E16 cochlear duct versus those from E12 cochlear duct. (**d**) Shows the numbers of differentially accessible peaks detected in Sox2-EGFP^*high*+^ cells at the indicated stages. Note that most significant changes were detected in the E12-14.5 window. (**e**) Shows differential motif enrichment in peaks increasing in accessibility in E12-16 Sox2-EGFP^*high*+^ cells from the cochlear duct relative to those decreasing in accessibility. (**f**) Plots normalized reads in the differentially accessible regions. (**g**) Plots the frequency of differentially enriched motifs relative to peak center.

To determine whether any of the peaks present in the Sox2-EGFP^high+^ cells were differentially accessible at different ages of cochlear development, we compared the ATAC-seq peaks from the three different ages using the differential accessibility analysis described above. We detected over 2,200 peaks in Sox2-EGFP^high+^ cells that change between E12 and E16. Many of the regions that show age-dependent differential accessibility are near regulatory genes, deafness genes and components of pathways of known significance to cochlear and prosensory development (Fig. 5a). For example, one peak  +268 kb from the *Atoh1* TSS increased significantly in accessibility in E14.5 when compared with E12 Sox2-EGFP^*high*+^ cells (*yellow* in Fig. 2g). The +268 kb differentially accessible region downstream of *Atoh1* is ~300 bp and contains putative binding sites for members of the Ets, MADS, Zf and Homeobox families as well as 5 consensus binding motifs for the bHLH transcription factor family (*not shown*).

Changes in cumulative accessibility in Sox2-EGFP^*high*+^ cells showed gene set enrichment for known processes in prosensory development. For example, at E14.5 versus E12, ‘positive regulation of synapse structure or activity’ was the most enriched process (p = 3.4e-4; Supplementary Fig. 8). The genes having the highest differential accessibility and therefore greatest contribution to the enrichment score for ‘positive regulation of synapse structure or activity’ in E14.5 versus E12 Sox2-EGFP^*high*+^ cells (i.e. the leading edge genes) included *Nrxn1, Adgrl2, Flrt2, Wnt7a, Bhlhb9* and *Nlgn1*. At E12 versus E14.5, ‘cochlear development’ was the most enriched process (p = 4.4e-4; Supplementary Fig. 8) and the leading edge genes included *Pax2*, *Fgfr3*, *Gli2* and *Wnt5a*.

Overall, accessibility in most differentially accessible peaks in Sox2-EGFP^*high*+^ cells diminished with developmental time (Fig. 5b,d,f; raw data in Supplementary Data 5). There was a greater difference in the number of differentially accessible peaks between E14.5 and E12 than between E14.5 and E16 in Sox2-EGFP^*high*+^ cells. Developmental changes in the cumulative accessibility of several genes including *Atoh1* and *Hes1* corresponded to known dynamics in gene expression^68–71^ (Fig. 5c).

Motif enrichment analysis of peak subsets that increased in accessibility across development showed significant enrichment for motifs of the Six, Rfx, Ctcf and Sox transcription factor families, as well as specific enrichment for some motifs not corresponding to any known regulators of cochlear development: Ebf and Nf1 (Fig. 5e,g, raw data in Supplementary Data 6). Motif enrichment analysis of peak subsets that decreased in accessibility across development also showed significant enrichment for motifs of the Six and Sox families as well as specific enrichment for Tead, Gata, Smad, Gli and Pax transcription factor families (Fig. 5e,g).

### Open chromatin regions in Sox2-EGFP^high+^ cells of the embryonic cochlear duct map to SNPs in human deafness genes

**Figure 6:**
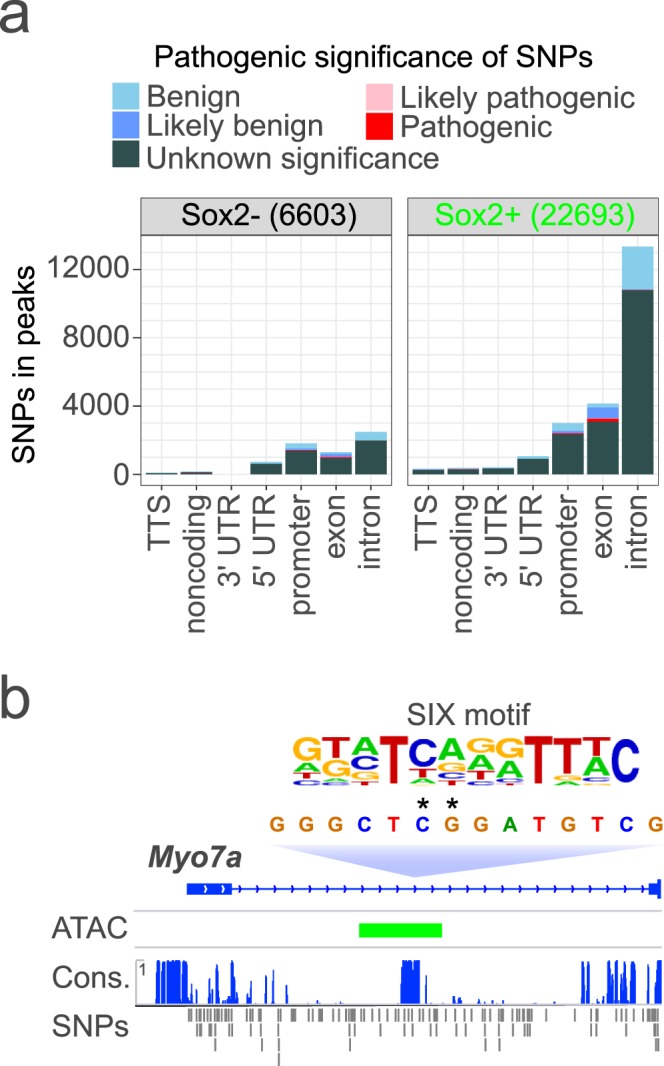
Human deafness gene SNPs in orthologous regions to ATAC-seq peaks detected in Sox2-EGFP^*high*+^ cells of the embryonic cochlear duct. Numbers of SNPs in the Deafness Variation Database (DVD) overlapping regions orthologous to ATAC-seq peaks in the indicated genomic features are shown in (**a**). Colors indicate pathogenicity as classified in the DVD. In (**b**), *asterisks* indicate two SNPs of unknown significance in *MYO7A* intron 1 that potentially affect binding at a SIX motif in a region of high evolutionary conservation (*Cons.)* that is orthologous to an ATAC-seq peak (*ATAC*) detected in *Myo7a* in embryonic mouse Sox2-EGFP^*high*+^ cochlear duct cells.

To determine whether open chromatin regions detected in Sox2-EGFP^*high*+^ cells of the embryonic cochlear duct in mouse have potential significance in the regulation of human deafness genes, mouse open chromatin regions were first mapped to the human genome (Hg19) using UCSC liftOver^72^. Variants in deafness-associated genes have recently been curated in the Deafness Variation Database (DVD)^22^. Over 48,000 of 65,129 ATAC-seq peaks detected in E12-16 Sox2-EGFP^*high+*^ cochlear duct cells mapped to regions of >70% sequence similarity in the human genome (Supplementary Data 8). Over 20,000 SNPs in the DVD overlap the open chromatin regions detected in mouse (Fig. 6a). Most SNPs in the DVD found to overlap with mouse open chromatin regions are intronic and of unknown significance to the pathogenesis of deafness (Fig. 6a). Some SNPs in human deafness genes coincide with transcription factor binding motifs in mouse open chromatin regions. For example, Fig. 6b shows two SNPs in a SIX motif overlapping an open chromatin region detected in Sox2-EGFP^*high*+^ cells of the embryonic mouse cochlear duct of high conservation within the first intron of *MYO7A*.

## Discussion

Our analysis of open chromatin in prosensory cells (Sox2-EGFP^*high*+^) adds considerable insight into gene regulation during early sensory development. During the development of the cochlea, hair cells are derived from Sox2^+^ progenitors in the prosensory domain of the floor of the cochlear duct^39,47,49,55,73^. Open chromatin regions detected in prosensory cells map to both experimentally validated and putative gene-activating regions (Fig. 2, Supplementary Fig. 6, Supplementary Data 2). Furthermore, over 29,000 open chromatin regions were found only in Sox2-EGFP^*high*+^ cochlear duct cells (Fig. 2g, Supplementary Data 1), suggesting a unique signature of open chromatin in these cells. By extension, we speculate that a unique epigenetic mechanism contributes to the specification of the prosensory lineage. Motif enrichment in prosensory open chromatin provides confirmatory evidence for the roles of Sox2, Six1 and Gata3^46–48^ in prosensory development (Fig. 3) and also implicates several transcription factor families in cochlear development that have not previously been reported to have a role in cochlear development, such as Tead, Ebf, Nfi, Klf/Sp and Ets families of transcription factors (Fig. 3). These findings imply that the regulatory factors identified may influence chromatin structure and cochlear cell reprogramming through pioneer activity^74^. In that regard, our analysis provides evidence for centering and increased density of transcription factor binding motifs in open chromatin peaks—two phenomena associated with pioneering activity^75^—of the transcription factor binding motifs enriched in prosensory open chromatin (Figs 3 and 4).

A recent report showed that Ctcf is required for cochlear development but not for prosensory gene expression^76^. Some previous evidence supports the possibility that Tead, Ebf, Nfi, Klf/Sp and Ets transcription factors could also influence cochlear development. For example, inhibition of the Yap–Tead interaction reduces supporting cell proliferation in the utricle^77^ and *yap1a* inhibition reduces neuromast formation in the zebrafish lateral line^78^, consistent with a possible role for the Hippo/Yap/Tead pathway in cochlear development. Previous findings in our lab showed that the inhibition of Fgf-signaling in cochlear explant cultures abolishes sensory specification as well as the expression of the downstream targets Etv4 and Etv5^34^, suggesting a role for the Ets family in sensory specification in the cochlea. Mutations in Klf/Sp, Ebf and Nfi family members have been associated with neurodevelopmental defects^79–88^. Furthermore, defects observed in *Nfix* mutants include hearing loss^84,86^. Our findings show enrichment of the Ebf1-4 motif in the open chromatin of Sox2-EGFP^*high*+^ cells (Fig. 3) and in regions that displayed an increase in accessibility in the Sox2-EGFP^*high*+^ cells between E12 and E16 (Fig. 4). Detailed transcriptomic analysis of isolated cochlear prosensory cells will be needed to further deduce the specific transcription factors of importance in prosensory development as each enriched motif corresponds to transcription factor families comprising multiple members.

Our findings provide insight into the epigenetic regulation of the prosensory cell during development. For example, accessibility in over 2,200 regions changed during development, largely between E12 and E14.5 and most dynamic sites diminished in accessibility. Single cell ATAC-seq has recently been developed^89^ and has the potential to better resolve the complexity of the developing prosensory population and the extent to which the early steps of hair cell and support cell specification relate to epigenetic dynamics in cochlear prosensory cells.

Findings in the present study add to knowledge of the epigenetic mechanisms of *Atoh1* regulation. Atoh1 is not only necessary for hair cell formation^56^ but also sufficient to induce hair cell differentiation in a limited population of cochlear cells^90^. Epigenetic regulation of *Atoh1* expression therefore has implications for hair cell regeneration strategies. Previous studies demonstrated a critical 3′ enhancer and showed association of this region as well as the promoter and an exonic region with activation-associated histone marks^26,91^. We find that these regions are accessible in Sox2-EGFP^*high*+^ cells of E12 cochlear duct, suggesting that the onset of *Atoh1* expression at E14.5^56^ is perhaps primed but unrelated to coincident changes in the accessibility of these regions. Rather, a + 268 kb distal region containing 5 bHLH binding motifs increased significantly in accessibility in E14.5 vs. E12 Sox2-EGFP^*high*+^ cells (Fig. 2g).

We were surprised to find greater cumulative accessibility in otic fibroblast marker genes such as *Coch*, Tbx18 and *Pou3f4* than in roof epithelial marker genes such as *Cldn11* and *Bsnd* (Fig. 2c) in Sox2-EGFP^−^ vs. Sox2-EGFP^*high*+^. Likewise, high expression of otic fibroblast markers was found in Lfng-GFP^−^, Pou4f3-GFP^−^ and Math1-GFP^−^ cells isolated in RNA-seq studies^92–94^. This could imply that all these GFP^−^ samples are enriched with otic fibroblasts. Otic fibroblasts and melanocytes are closely associated with the roof of the cochlear duct at prosensory stages^95,96^ and we therefore speculate that the enrichment for otic fibroblasts—despite microdissection away of most surrounding mesenchyme in these samples—could be related to the relative efficiency of dissociating mesenchymal cells versus epithelial cells. An alternative explanation for the otic fibroblast gene expression and accessibility evident in these samples is that fibroblasts or mesenchymally-derived epithelial cells are perhaps in the cochlear duct. Lineage tracing using the Wnt1-CreER demonstrated neural crest contributions to the cochlear duct epithelium in one study^97^, but not in a more recent study using a different reporter^98^. Regardless, it is possible that the FACS sorted cells contain otic fibroblasts even though the majority of these cells were removed by microdissection.

Most sensorineural hearing loss relates to the loss or dysfunction of hair cells and most emerging therapies for hearing loss therefore seek to protect or replace hair cells^99^. Functionally significant regeneration of cochlear hair cells is not robust in mammalian models of hearing loss. The strategies showing greatest promise for the regeneration of hair cells have altered the activity of Sox2, Atoh1 and other transcription factors to achieve the conversion of limited populations of cochlear cells to hair cells^35,90,100–107^ and also the differentiation vestibular-like hair cells from embryonic stem cells^108,109^. Findings from our study implicate new transcription factor classes in prosensory development that merit consideration as possible regulators of support cell and hair cell formation.

In summary, ATAC-seq analysis of Sox2-EGFP^*high*+^ cells of the embryonic cochlear duct implicates novel classes of transcription factors in inner ear development and provides strong evidence for a prosensory-specific landscape of open chromatin as well as epigenetic dynamics during prosensory cell development. These datasets (in Supplementary Data 1, 3, 5 and 8 and summarized in Supplementary Fig. 9) not only provide insights into the gene regulatory networks guiding prosensory development in the cochlea but also represent a significant resource for the mechanistic study of the *cis*-regulation of deafness genes and genes of interest for regeneration studies in the inner ear.

## Materials and Methods

### Mice

Sox2-EGFP knockin mice^59^ from (Jackson Stock: 017592) were bred to generate E12-16 litters. Mice were housed in the University of Washington Department of Comparative Medicine. All procedures were reviewed and approved by the Institutional Animal Care and Use Committee of the University of Washington and performed in accordance with NIH guidelines. Sox2-EGFP^+^ embryos were identified by epifluorescence. Stages were verified by Theiler’s criteria.

### Vibratome sections of Sox2-EGFP^+^ embryonic temporal bone

E12-16 Sox2-EGFP^+^ heterozygous embryos were decapitated in Hank’s buffered salt solution (Cat. No. 14025-92; Thermo Fisher Scientific; Waltham, MA) and fixed overnight at 4 °C in 4% paraformaldehyde (Cat. No. 15710; Electron Microscopy Sciences; Hatfield, PA) in phosphate-buffered saline (PBS; Cat. No. BP399; Thermo Fisher Scientific). After 3 × 30 minute washes in PBS, temporal bones were isolated by microdissection, embedded in in 4% agarose and sectioned at 100 μm with a vibratome.

### Sox2 and EGFP immunolabeling

Sections were immunolabeled essentially as described by Hartman *et al*.^110^. Sections were permeabilized (2% Triton-X-100/PBS) for 30 minutes, then blocked (10% donkey serum/0.5% Triton-X-100/PBS) for 2–4 hours. Sections were incubated overnight in 1:200 goat anti-Sox2 (Y-17; Santa Cruz) and 1:250 chick anti-GFP (ab13970; Abcam) diluted in block. After 3 × 1 hour washes in 0.5% Triton-X-100/PBS, sections were incubated overnight in block, then overnight in 1:400 Alexa488-conjugated donkey anti-goat and 1:400 Alexa647-conjugated donkey anti-chick diluted in block. After 3 × 1 hour washes in 0.5% Triton-X-100/PBS, sections were cleared by 3 × 10 minute incubations in 60% glycerol/PBS, then imaged.

### Confocal microscopy

Z-stacks of Sox2 and EGFP immunofluorescence were collected on a LSM880 with Airyscan using 10X/0.45 (1.1 μm steps) and 20X/0.8 M27 (0.3 μm steps) Plan-Apochromat objectives (Ziess, Jena, Germany). Zeiss Zen Black v14.0.0.0 was used to acquire micrographs and to perform Airyscan processing, maximum intensity projections (i.e. of 2–5 optical sections) and linear adjustments to black and white points.

### Isolation of Sox2-EGFP^high+^ cochlear duct cells

Cochlear ducts were dissected away from mesenchyme after collagenase digestion as described previously^34^. Care was taken to dissect away all cochlear nerve tissue to remove the potentially confounding population of Sox2-EGFP^+^ glial cells. Cochlear duct cells were then dissociated using papain at 37 °C and sorted to isolate live Sox2-EGFP^*high*+^ cells using a BD Aria III (85 μm nozzle, flow rate 1–3) as shown in Fig. 1a. All dissection, sorting, spins and washes were performed in ice-cold media. For comparative ATAC-seq analysis, Sox2-EGFP^−^ cells were isolated in parallel in some cases. Gating was centered on the highest and lowest peaks in the histogram and EGFP^low^ cells were excluded.

### ATAC-seq

Sox2-EGFP^*high*+^ cells were isolated from 2 litters per embryonic stage (i.e. 1 litter per biological replicate of 6–12 pooled cochlear ducts at E12, E14.5 and E16). ATAC-seq sample preparation, transposition and library amplification were performed as described by Buenrostro and colleagues^111^. Briefly, adapters for library amplification and sequencing were inserted into open chromatin via transposase *in vitro* and libraries were prepared using the Illumina Nextera kit (Cat. No. FC-121-1030). The Genomics & Bioinformatics Shared Resource at the Fred Hutchinson Cancer Research Center (Illumina Hi-seq 2500) and Seattle Genomics (Illumina Next Seq. 500) performed deep sequencing. Adapters were trimmed from reads and low-quality sequences (Phred <33) were removed using Trim Galore!^112,113^. Reads were aligned to mm9 using Bowtie2^114^ (option:–very-sensitive). Duplicate reads were marked using Picard ‘MarkDuplicates’ (http://broadinstitute.github.io/picard/). Duplicate and mitochondrial reads were removed using SAMtools^115^. Bigwigs were generated using a custom script (https://rpubs.com/achitsaz/98857) and visualized in IGV^116^. Supplementary Table 1 describes samples and coverage. Percentages of reads in peaks were found using ‘plotEnrichment’ in deepTools2^117^. ATAC-seq data has been deposited in NCBI GEO database under the accession code GSE131775.

### Peak calling and filtering

Peaks were called using MACS2^60^ (options:–nomodel–shift -100–extsize 200). Irreproducible peaks were filtered using BEDOPS^118^ (command: ‘bedops -e 1 replicate1.bed replicate2.bed’). Overlapping and nearby ATAC-seq peaks were merged using BEDtools^119^ (command: bedtools merge -i replicatedpeaks.bed -d 100). Peaks in regions previously found by ENCODE to produce artefactual signal^120^ (http://mitra.stanford.edu/kundaje/akundaje/release/blacklists/) were removed using BEDOPS command: -n 1. Only reproducible peaks (i.e. peaks replicated in at least one other dataset of the same sample type) were used for genomic annotation, motif enrichment and other analysis. To find prosensory-specific peaks, BEDOPS^118^ (command: -n 1) was used to isolate peaks of Sox2-EGFP^*high*+^ cochlear duct cells not overlapping with any ATAC-seq peaks called as described above in Sox2-EGFP^−^ cochlear duct cells as well as in the following ENCODE ATAC-seq datasets: G1E mouse embryonic stem cells (ENCSR280ZDP), E14.5 C57Bl/6 mouse facial prominence (ENCSR876SYO), forebrain (ENCSR810HQR), midbrain (ENCSR384JBF), hindbrain (ENCSR798FDL), neural tube (ENCSR700QBR), limb (ENCSR460BUL), kidney (ENCSR758IRM), intestine (ENCSR150EOO), liver (ENCSR032HKE), lung (ENCSR335VJW), stomach (ENCSR618HDK) and heart (ENCSR068YGC). ENCODE datasets were aligned and peaks were called using the pipeline described above.

### Annotation and genomic feature enrichment analysis

Annotation of ATAC-seq peaks and genomic annotation enrichment analysis were performed using HOMER^121^, which assigns peaks to the nearest TSS then annotates peaks based on UCSC refGene annotations^122^ for mm9.

### Curation of previously reported otic enhancers and promoters

To determine whether ATAC-seq detects known otic enhancers, we mapped ATAC-seq peaks to known otic enhancers. As no comprehensive list for otic enhancers and promoters driving gene-activation *in vivo* existed, a list had to be generated through a comprehensive review. To generate the list, we considered our knowledge, entries in the VISTA Enhancer Browser^61^ listing ‘ear’ expression patterns as well as all articles published up to March 1, 2018 found using the Pubmed search: (“ear”[MeSH Terms]) AND regulatory sequences, nucleic acid[MeSH Terms]. This research yielded 56 otic enhancers and promoters described in sufficient detail to map to mm9 and determine overlap with ATAC-seq data. Regions identified in other systems (e.g. human cell lines, chick and zebrafish) were mapped to orthologous regions in mm9 using BLAST and liftOver. The list is available in Supplementary Data 2.

### Differential accessibility analysis in ATAC-seq peaks

Differential accessibility across ATAC-seq sample groups was determined as detailed in the edgeR^62^ users guide. To define the regions for differential accessibility analysis, coordinates of all overlapping and nearby (<100 bp) intervals of replicated ATAC-seq peaks detected in all compared groups was determined using ‘merge’ in BEDtools^119^ (option: -d 100). A matrix of counts per million for all samples was then generated using the ‘featureCounts’ in the Rsubread package (options: isPairedEnd = TRUE, maxFragLength = 2000). The counts matrix was filtered to select rows having at least 1 count per million in *n* − 1 samples to minimize the influence of variability at the threshold for sensitivity on the analysis. Cumulative fold differences in accessibility at each gene were calculated as the sum of the fold differences of all peaks nearest to each gene.

### Correlation analysis of ATAC-seq reads in peaks

Using deepTools2^117^, matrix of read counts in each sample was first computed using ‘multiBamSummary‘. A Spearman correlation matrix of reads in peaks was then generated ‘plotCorrelation’. A distance matrix for the correlations was generated using ‘dist’ (https://www.rdocumentation.org/packages/stats/versions/3.5.2; mode = Euclidean). Clustering of the correlation matrix was performed in RStudio using ‘hclust’ in the fastCluster package^123^ (mode = Ward.D2). A heatmap of the correlation matrix was generated using ‘heatmap.2’ (https://www.rdocumentation.org/packages/gplots).

### Intersect analysis of ATAC-seq and Methyl-seq data

The ‘venn’ command in Intervene^124^ was used to calculate the intersect sizes for ATAC-seq peak and Methyl-seq regions and plot the venn diagrams.

### Motif-enrichment analysis

Motif-enrichment analysis in ATAC-seq peaks was performed using ‘findmotifsgenome.pl’ in HOMER command (options: -size given -mask) and the HOMER library of consensus binding motifs determined *in vivo* by ChIP-seq. Frequencies of motifs per base pair per peak (Fig. 3b) were calculated using ‘annotatepeaks.pl’ in HOMER.

### Correlation analysis of motif matrices

To group similar motifs in Fig. 3a, ‘comparemotifs.pl’ in HOMER was used to generate a correlation matrix of the motif matrices in the HOMER motif library. Clustering of the correlation matrix and plotting was performed as described above.

### Co-occurrence of motifs in ATAC peaks

The co-occurrence of significantly enriched motifs (Fig. 4c and Data 7) was calculated using ‘annotatepeaks.pl’ in HOMER. Clustering of the co-occurrence ratio matrix and plotting was performed as described above.

### Gene set enrichment analysis

Gene set enrichment analysis^125^ for GO Biological Processes (http://www.go2msig.org/cgi-bin/prebuilt.cgi?taxi=10090) was performed on the cumulative fold differences in accessibility using the fgsea package^126^ (options: minSize = 25, maxSize = 500, nperm = 10000). Enriched gene sets were reduced to independent gene sets using ‘collapsePathways’ in fgsea.

### Mapping mouse ATAC-seq peaks to the human genome

The UCSC liftOver^127^ command line tool was used to convert mm9 coordinates of ATAC-seq peaks to orthologous regions in the human genome Hg19 (option: -minMatch = 0.7). The human SNPs in the Deafness Variation Database v8.1 (http://deafnessvariationdatabase.org/public/) overlapping the conserved ATAC-seq regions were identified using ‘findOverlaps’ in the GRanges^128^ package. Transcription factor binding motifs in the conserved regions were visualized in IGV using the file available at http://homer.ucsd.edu/homer/index.html.

### Plotting and other statistical analysis

Cumulative accessibility was plotted in Prism v5.0 f. For Fig. 4f, a counts matrix was generated using ‘computeMatrix’, then plotted using ‘plotHeatmap’ in deepTools2^117^. Venn diagrams were plotted using Intervene^124^. Other plots were generated in RStudio v1.1.456. Scatterplots, bar plots and line plots were generated using the ggplot2 package^129^. Correlation heatmaps were generated using the gplots package (https://www.rdocumentation.org/packages/gplots). Figure compositions were performed in Adobe Illustrator and Photoshop CC 2017 (San Jose, CA). To test for differences in cell counts across development, one-way ANOVA and Tukey’s HSD multiple comparisons tests were performed in R.

## Supplementary information

Supplementary figures and legends

Supplementary data 1

Supplementary data 2

Supplementary data 3

Supplementary data 4

Supplementary data 5

Supplementary data 6

Supplementary data 7

Supplementary data 8
