# Supplementary material for: Open chromatin dynamics in prosensory cells of the embryonic mouse cochlea
Source: Sci Rep. 2019 Jun 21;9:9060. doi: 10.1038/s41598-019-45515-2 (PMC6588700; doi:10.1038/s41598-019-45515-2)
Supplement: Supplementary file 1 — Supplementary figures and legends [file 41598_2019_45515_MOESM1_ESM.pdf]

SUPPLEMENTARY INFORMATION

Open chromatin dynamics in prosensory cells of the embryonic mouse cochlea

Brent A. Wilkerson[1,2], Alex D. Chitsazan[1,2,3], Leah S. VandenBosch[1,4], Matthew S. Wilken[1,5], Thomas Reh[1,2], Olivia Bermingham-McDonogh[1,2,*]

Supplementary figures 1-9

Supplementary table 1

Supplementary data 1-8

1. Department of Biological Structure, University of Washington, Box 357420 Seattle, WA, 98195, USA
2. Institute for Stem Cells and Regenerative Medicine, University of Washington, Washington, USA
3. Department of Biochemistry, University of Washington, Seattle, WA, USA
4. Molecular and Cellular Biology Program, University of Washington, Seattle, WA, USA
5. Altius Institute for Biomedical Sciences, Seattle, WA, USA
* oliviab@uw.edu

Supplementary figure 1. p75/Ngfr immunofluorescence in E16.5 Sox2-EGFP cochlea. p75/Ngfr immunofluorescence (*magenta*) in Claudius cells delimits Sox2-EGFP expression (*arrow*) and also demonstrates pillar cells (center) and cochlear nerve fibers (top left). Bars represent 100 μm.

Supplementary figure 2. Sox2-EGFP expression in prosensory cells of the embryonic vestibular system. Immunofluorescence of Sox2-EGFP expression (*green*) and of endogenous Sox2 (*magenta*) is shown in vibratome sections of otolith organs (**a**, **d** and **e**) and of cristae (**b**, **d** and **e**). Note that Sox2-EGFP expression corresponds to Sox2 immunofluorescence in E12.5-16.5 maculae and cristae. The localization of Sox2-EGFP expression shown here is representative of that in at least three temporal bones. Bars represent 100 μm. *sa*, saccule; *ut*, utricle

Supplementary figure 3. Sox2-EGFP expression relative to endogenous Sox2 in E16 prosensory cells. Immunofluorescence of Sox2-EGFP expression (*green*), Myosin 7a (*white*) and of endogenous Sox2 (*magenta*) are shown in vibratome sections of E16 cochlea (**a**) and crista (**b**). Note that reduced Sox2 immunofluorescence in some hair cells indicated by arrows corresponds to decreased Sox2-EGFP. Bar represents 50 μm. *co*, cochlea; *cr*, crista

Supplementary figure 4. Cell counts in the embryonic cochlear duct. Bars indicate mean cell numbers per microdissected cochlear duct (**a**) and the mean percentage of Sox2-EGFP^{high+} cells per cochlear duct (**b**) in pooled samples of cochlear ducts. A subset of samples graphed in (**a**) are graphed in (**b**). (**a**) includes additional samples counted using a hemocytometer not analyzed by flow cytometry. Labels in (**b**) indicate the numbers of cochlear ducts pooled per sample analyzed by ATAC-seq.

ANOVA p-values were p = 0.000622 and p = 0.00275 for (**a**) and (**b**), respectively. Significant p-values from Tukey multiple comparisons are shown.

Supplementary figure 5. Cochlear ATAC-seq peak overlap with unmethylated regions and low methylated regions of the sensory epithelium. (**a**) shows overlaps of ATAC-seq peaks in E14.5-16 Sox2-EGFP^{-} cochlear duct cells, ATAC-seq peaks in E12-16 Sox2-EGFP^{high+} cochlear duct cells, low methylated regions E16.5 cochlear sensory epithelium and unmethylated regions E16.5 cochlear sensory epithelium. Note that most ATAC-seq peaks in cochlear duct cells correspond to UMRs and LMRs in sensory epithelium but many LMRs in sensory epithelium do not correspond to ATAC-seq peaks in cochlear duct cells. (**b**) shows overlap of differentially accessible regions in E12-16 Sox2-EGFP^{high+} cochlear duct cells and differentially methylated regions in E16.5-P0 cochlear sensory epithelium.

Supplementary figure 6. Enrichment of embryonic cochlear duct Sox2-EGFP^{high+} ATAC-seq in putative gene regulatory regions. Bars indicate log2 fold enrichment of bp in E12-16 cochlear Sox2-EGFP^{high+} ATAC-seq peaks (i.e. all replicated peaks) mapping to genomic features relative to the total bp of respective genomic features.

Supplementary figure 7. Correlation matrix of the HOMER library of known transcription factor binding motifs. The heatmap shows Pearson correlations of HOMER motif matrices clustered based on similarity. The clusters of highly correlated motifs corresponding to several transcription factor families are indicated in the labels on the right.

Supplementary figure 8. Gene Set Enrichment Analysis of differentially accessible ATAC-seq peaks in E12-16 Sox2-EGFP^{high+} cochlear duct cells. Results from Gene Set Enrichment Analysis of the cumulative differential accessibility i.e. the sum of fold differences of all ATAC-seq peaks nearest to each gene) in Sox2-EGFP^{high+} cells isolated from the embryonic cochlear duct E14.5 vs. E12 and at E16 vs. E14.5 show differentially enriched biological processes and the distribution of gene ranks, the normalized enrichment score (NES) and the p-value.

Supplementary figure 9. Summary of ATAC-seq peak subsets. The relationships of the differentially-accessible and differentially-detected peak subsets are summarized in **a-d**. Peak subsets are available as a resource in the **Supplementary data**.

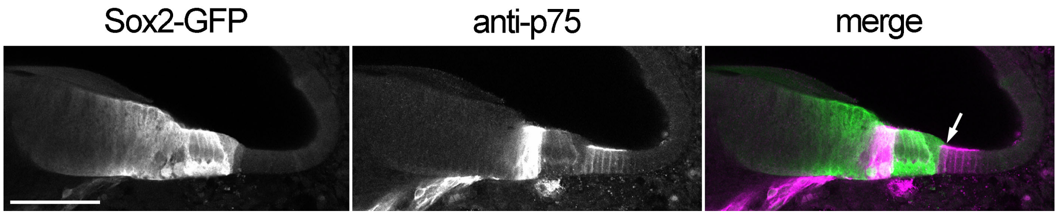
Sox2-GFP
anti-p75
merge

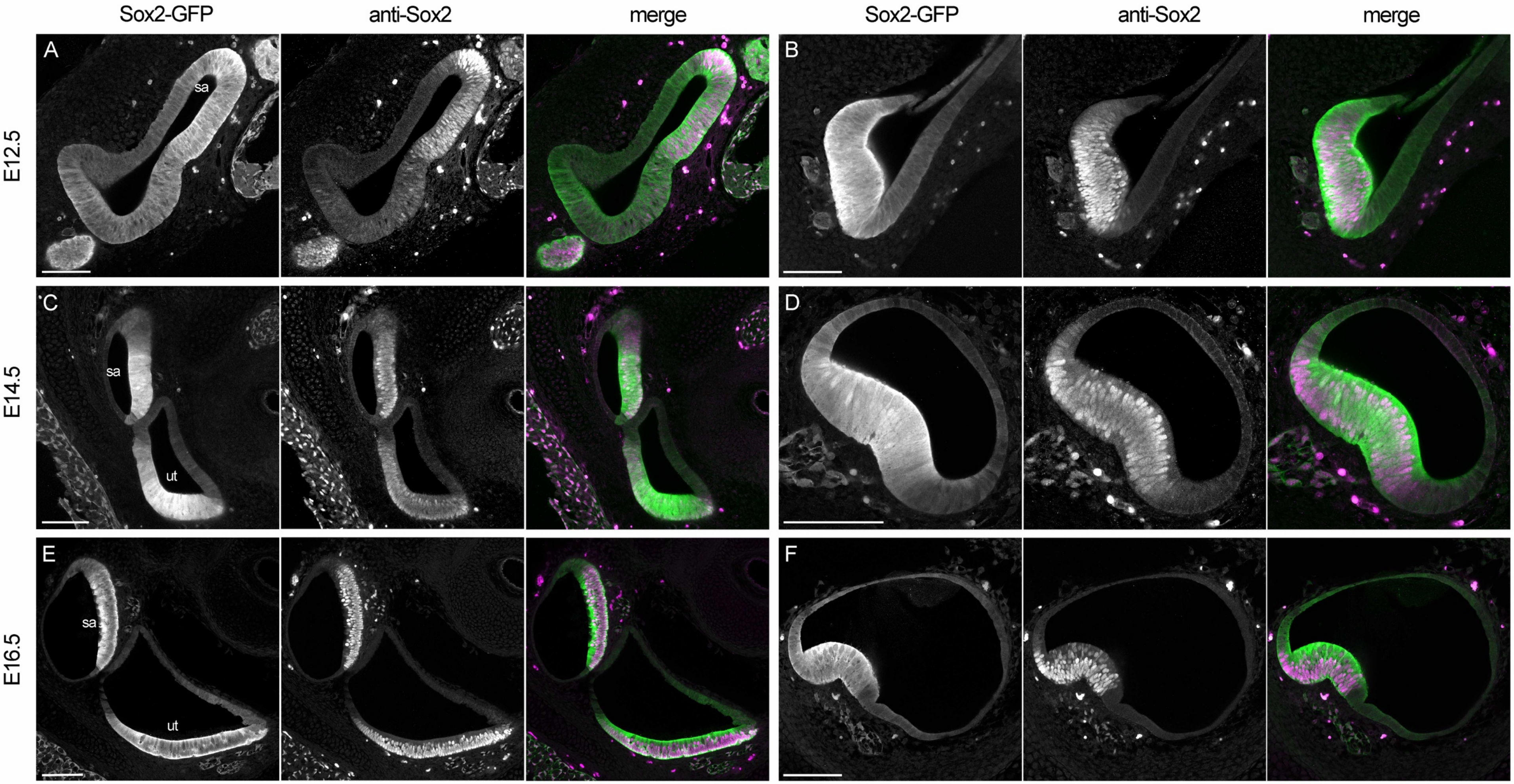
Sox2-GFP
anti-Sox2
merge
Sox2-GFP
anti-Sox2
merge
E12.5
E14.5
E16.5
A
B
C
D
E
F
sa
sa
ut
sa
ut

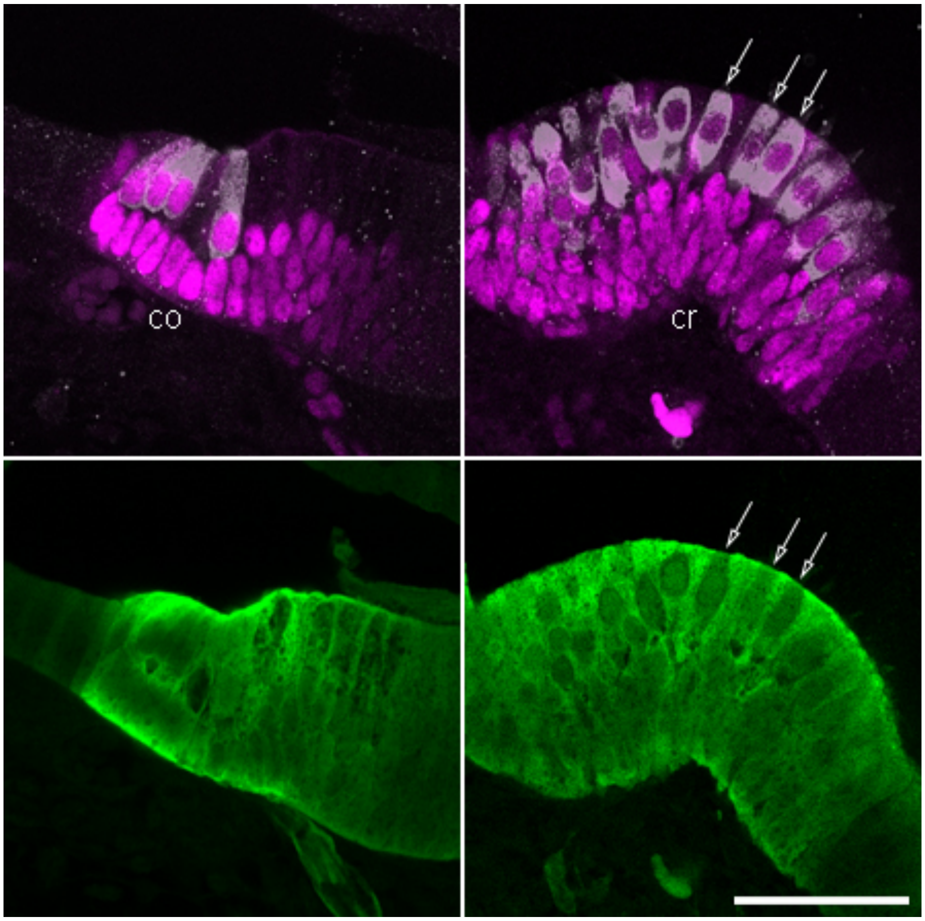
co
cr

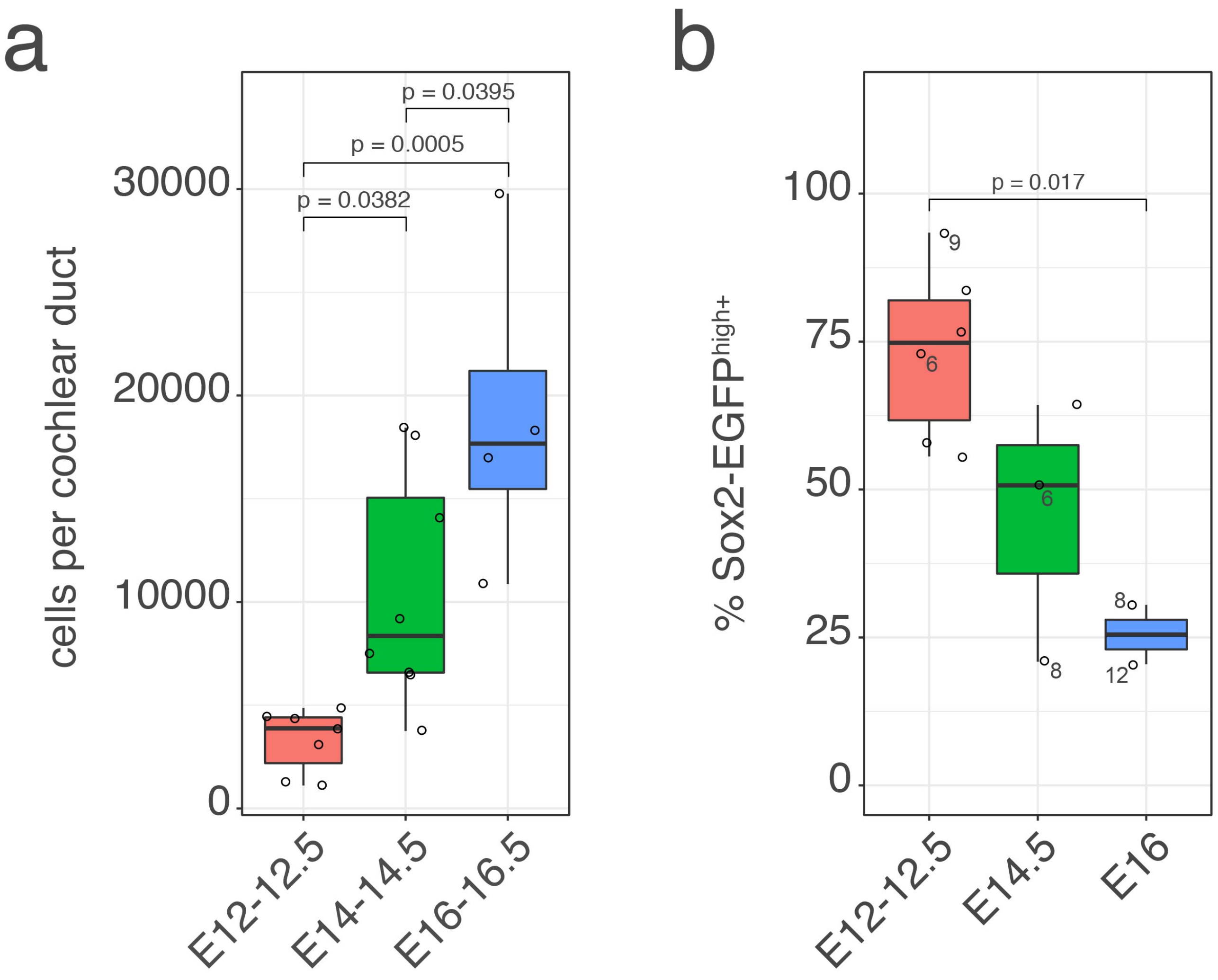

a
p = 0.0395
p = 0.0005
p = 0.0382
30000
20000
10000
0
cells per cochlear duct
E12-12.5
E14-14.5
E16-16.5
b
p = 0.017
100
75
50
25
0
% Sox2-EGFP^high+
9
6
6
8
8
12
E12-12.5
E14.5
E16

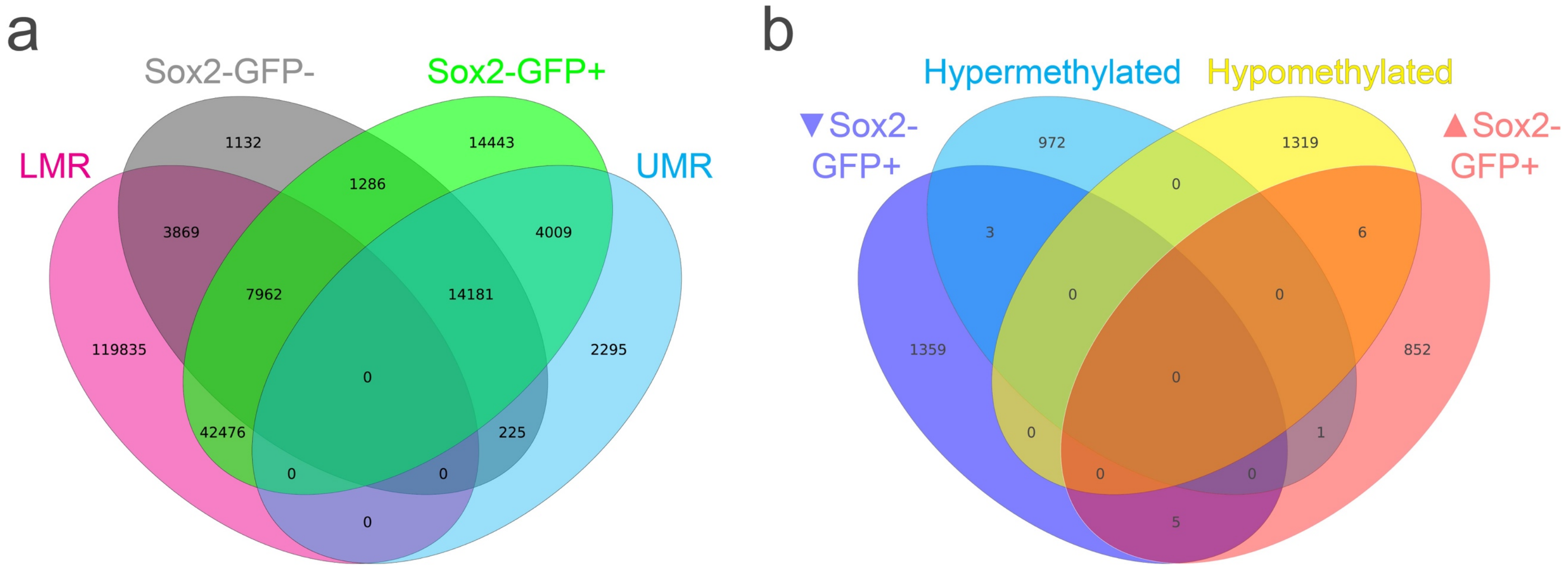
a
LMR
Sox2-GFP-
Sox2-GFP+
UMR
1132
14443
1286
3869
4009
7962
14181
119835
2295
0
42476
225
0
0
0
b
▼Sox2-GFP+
Hypermethylated
Hypomethylated
▲Sox2-GFP+
972
1319
0
3
6
0
0
1359
852
0
0
1
0
0
5

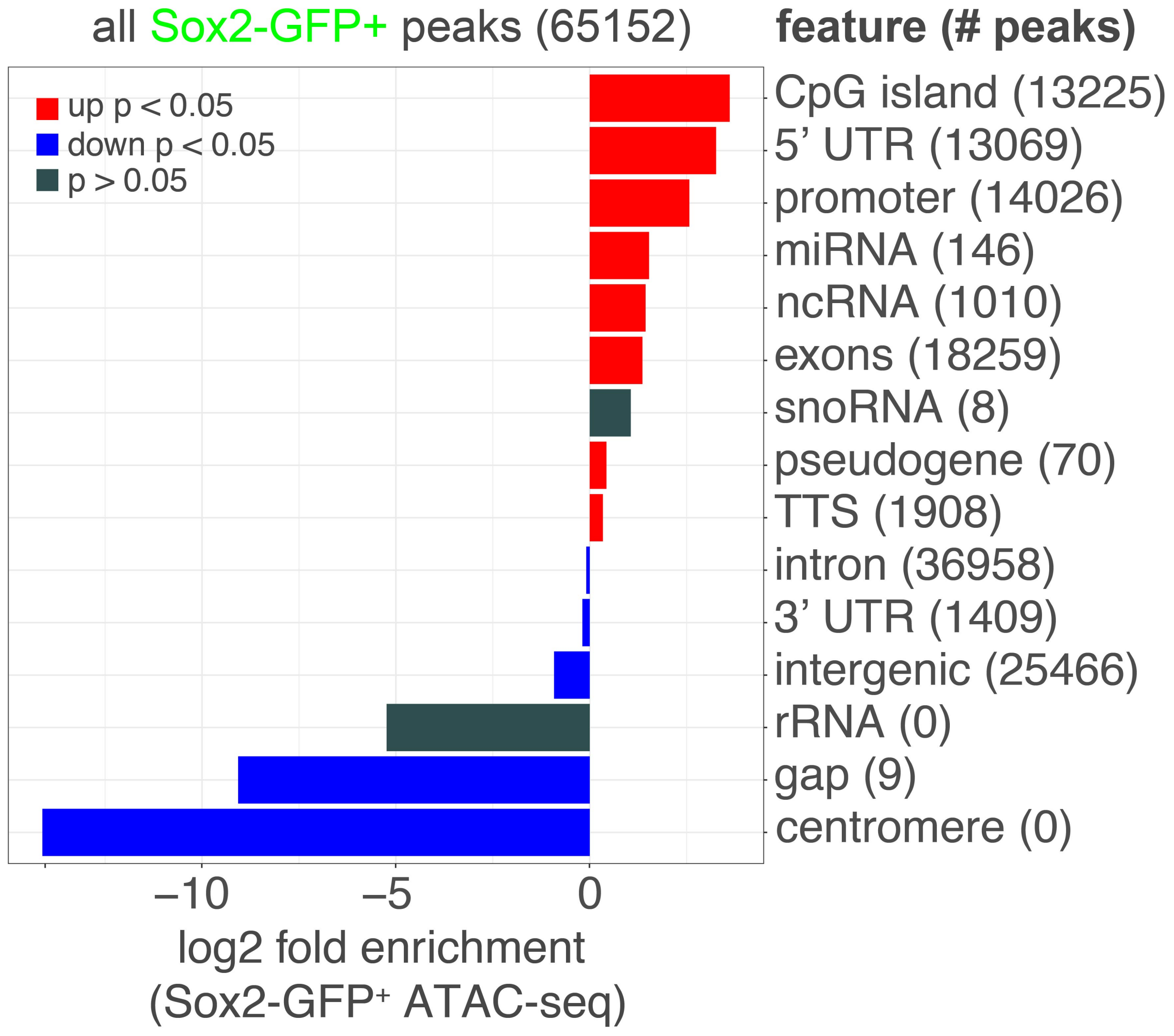

all Sox2-GFP+ peaks (65152)
feature (# peaks)
up p < 0.05
down p < 0.05
p > 0.05
CpG island (13225)
5’ UTR (13069)
promoter (14026)
miRNA (146)
ncRNA (1010)
exons (18259)
snoRNA (8)
pseudogene (70)
TTS (1908)
intron (36958)
3’ UTR (1409)
intergenic (25466)
rRNA (0)
gap (9)
centromere (0)
−10
−5
0
log2 fold enrichment
(Sox2-GFP+ ATAC-seq)

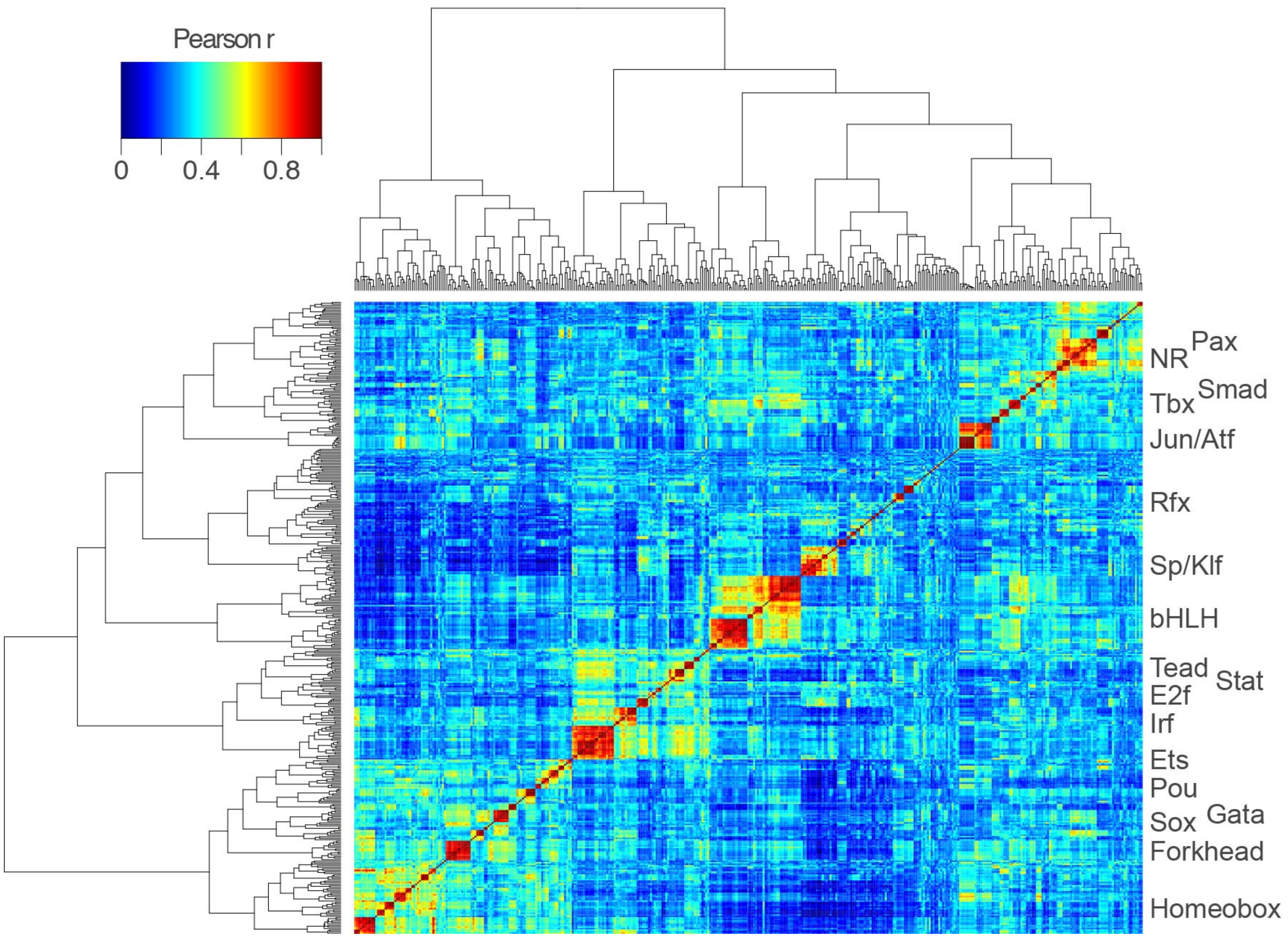
Pearson r
0
0.4
0.8
NR
Pax
Tbx
Smad
Jun/Atf
Rfx
Sp/Klf
bHLH
Tead
Stat
E2f
Irf
Ets
Pou
Sox
Gata
Forkhead
Homeobox

E14.5/E12

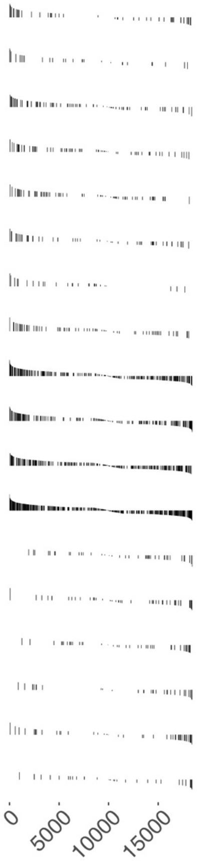

Process	NES	p-value
positive regulation of synapse assembly	1.86	1.2e−04
reproductive behavior	1.80	2.5e−03
microtubule based movement	1.74	5.6e−04
carbohydrate biosynthetic process	1.65	3.1e−03
blood coagulation	1.63	4.1e−03
negative regulation of cell migration	1.60	1.1e−02
ribonucleoprotein complex subunit organization	1.60	1.7e−02
negative regulation of immune response	1.56	1.2e−02
actin filament based process	1.50	1.1e−03
kidney development	1.45	6.6e−03
regulation of kinase activity	1.38	1.3e−02
cellular response to endogenous stimulus	1.29	1.8e−02
internal peptidyl lysine acetylation	−1.76	4.5e−03
neuroblast proliferation	−1.77	3.8e−03
photoreceptor cell development	−1.85	2.4e−03
midbrain development	−1.95	1.3e−03
cell differentiation in spinal cord	−2.07	5.5e−04
cochlea development	−2.07	8.9e−04

E16/E14.5

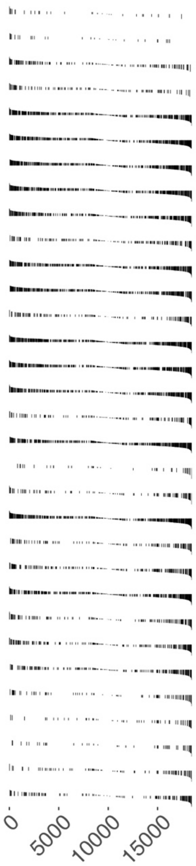

Process	NES	p-value
cellular response to metal ion	1.97	1.1e−03
regulation of cell matrix adhesion	1.93	1.7e−03
regulation of membrane potential	1.58	1.6e−03
organophosphate biosynthetic process	1.58	2.8e−03
▼ transcription from RNA polymerase II promoter	−1.49	6.4e−04
brain development	−1.57	1.1e−04
regulation of cell development	−1.58	1.1e−04
negative regulation of cell differentiation	−1.60	1.1e−04
epithelial cell differentiation	−1.64	1.1e−04
sex differentiation	−1.67	1.1e−03
regulation of anatomical structure morphogenesis	−1.68	1.1e−04
positive regulation of cell differentiation	−1.68	1.1e−04
Rho protein signal transduction	−1.68	7.5e−04
embryonic morphogenesis	−1.72	1.1e−04
morphogenesis of an epithelium	−1.75	1.1e−04
neuron projection morphogenesis	−1.75	1.1e−04
cardiac muscle tissue development	−1.75	2.5e−04
enzyme-linked receptor protein signaling pathway	−1.76	1.1e−04
lung epithelium development	−1.83	1.6e−03
mesenchymal cell differentiation	−1.84	3.9e−04
cell morphogenesis involved in differentiation	−1.88	1.1e−04
regulation of organ morphogenesis	−1.91	1.2e−04
urogenital system development	−1.93	1.2e−04
skeletal system development	−1.93	1.1e−04
axon guidance	−1.93	1.2e−04
ear development	−1.95	1.2e−04
cellular response to growth factor stimulus	−1.95	1.2e−04
kidney epithelium development	−1.97	1.3e−04
organ formation	−1.98	1.4e−04
regulation of morphogenesis of a branching structure	−2.07	1.4e−04
digestive system development	−2.09	1.3e−04
cartilage development	−2.15	1.3e−04

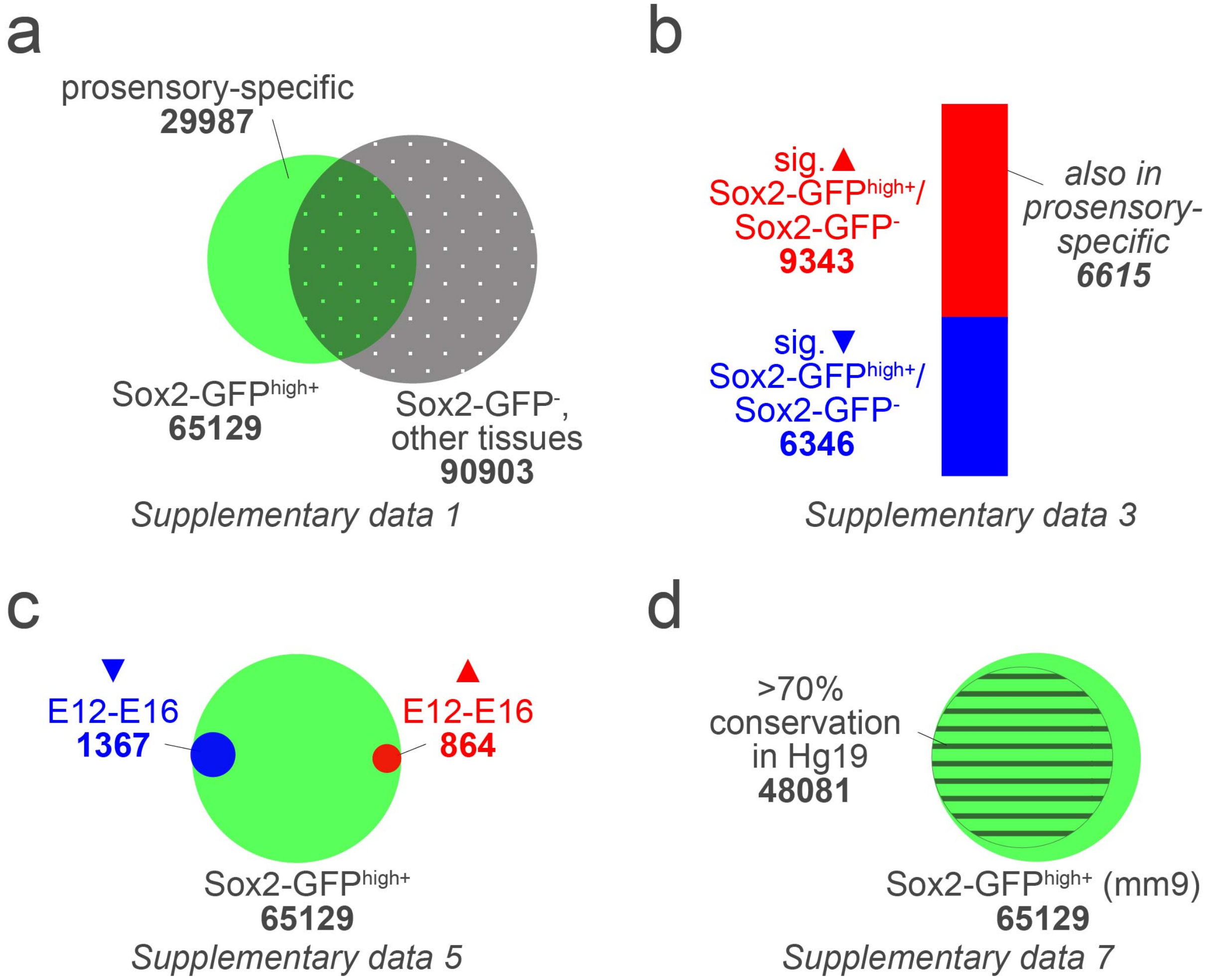
a
prosensory-specific
29987
Sox2-GFPhigh+
65129
Sox2-GFP-,
other tissues
90903
Supplementary data 1
b
sig. ▲
Sox2-GFPhigh+/
Sox2-GFP-
9343
also in
prosensory-
specific
6615
sig. ▼
Sox2-GFPhigh+/
Sox2-GFP-
6346
Supplementary data 3
c
▼
E12-E16
1367
▲
E12-E16
864
Sox2-GFPhigh+
65129
Supplementary data 5
d
>70%
conservation
in Hg19
48081
Sox2-GFPhigh+ (mm9)
65129
Supplementary data 7

Supplementary table 1. Cochlear duct ATAC-seq sample descriptions.

Stage	Sox2-GFP	replicate	sequencing mode	sequencing batch	reads	peaks	% reads in peaks
E12	+	1	paired-end	2	10653165	14354	6.5
E12	+	2	paired-end	2	11532663	8036	3.0
E14.5	+	1	paired-end	2	48117323	112999	50.3
E14.5	+	2	paired-end	2	63836522	26730	4.9
E14.5	-	2	paired-end	2	75726632	28903	6.2
E16	+	1	paired-end	1	44955094	39890	16.7
E16	+	2	paired-end	2	28444425	72846	26.3
E16	-	1	paired-end	1	30612005	29264	12.5
E16	-	2	paired-end	2	41769170	29966	8.3

Supplementary data 1. Coordinates for ATAC-seq peaks in E12-16 Sox2-EGFP^{high+} cochlear duct cells, ATAC-seq peaks in E14.5-16 Sox2-EGFP^{-} cochlear duct cells and ATAC-seq peaks detected only in Sox2-EGFP^{high+} cochlear duct cells.

Supplementary data 2. Coordinates for reported otic regulatory elements and ATAC-seq peak overlap.

Supplementary data 3. Differential accessibility in Sox2-EGFP^{high+} vs. Sox2EGFP^{-} cochlear duct cells.

Supplementary data 4. Motif enrichment analysis of ATAC-seq peaks in Sox2-EGFP^{high+} cochlear duct cells, Sox2EGFP^{-} cochlear duct cells and ATAC-seq peaks detected only in Sox2-EGFP^{high+} cochlear duct cells.

Supplementary data 5. Differential accessibility in E12-16 Sox2-EGFP^{high+} cochlear duct cells.

Supplementary data 6. Motif enrichment analysis of differentially accessible ATAC-seq peaks in Sox2-EGFP^{high+} cochlear duct cells.

Supplementary data 7. Motif co-occurrence in prosensory-specific open chromatin regions of the embryonic cochlear duct.

Supplementary data 8. Human genome Hg19 coordinates of regions orthologous to ATAC-seq peaks detected in embryonic mouse Sox2-EGFP^{high+} vs. Sox2EGFP^{-} cochlear duct cells.
